# Nebulized anticoagulants for acute lung injury - a systematic review of preclinical and clinical investigations

**DOI:** 10.1186/cc11325

**Published:** 2012-04-30

**Authors:** Pieter R Tuinman, Barry Dixon, Marcel Levi, Nicole P Juffermans, Marcus J Schultz

**Affiliations:** 1 Department of Intensive Care Medicine and Laboratory of Experimental Intensive Care and Anesthesiology (L·E·I·C·A), Academic Medical Center, Meibergdreef 9, Amsterdam, 1105 AZ, The Netherlands; 2 Department of Intensive Care, St Vincent's Hospital, 41 Victoria Parade, Fitzroy, Melbourne, VIC 3065, Australia; 3 Department of Internal Medicine, Academic Medical Center, Meibergdreef 9, Amsterdam, 1105 AZ, The Netherlands

## Abstract

**Background:**

Data from interventional trials of systemic anticoagulation for sepsis inconsistently suggest beneficial effects in case of acute lung injury (ALI). Severe systemic bleeding due to anticoagulation may have offset the possible positive effects. Nebulization of anticoagulants may allow for improved local biological availability and as such may improve efficacy in the lungs and lower the risk of systemic bleeding complications.

**Method:**

We performed a systematic review of preclinical studies and clinical trials investigating the efficacy and safety of nebulized anticoagulants in the setting of lung injury in animals and ALI in humans.

**Results:**

The efficacy of nebulized activated protein C, antithrombin, heparin and danaparoid has been tested in diverse animal models of direct (for example, pneumonia-, intra-pulmonary lipopolysaccharide (LPS)-, and smoke inhalation-induced lung injury) and indirect lung injury (for example, intravenous LPS- and trauma-induced lung injury). Nebulized anticoagulants were found to have the potential to attenuate pulmonary coagulopathy and frequently also inflammation. Notably, nebulized danaparoid and heparin but not activated protein C and antithrombin, were found to have an effect on systemic coagulation. Clinical trials of nebulized anticoagulants are very limited. Nebulized heparin was found to improve survival of patients with smoke inhalation-induced ALI. In a trial of critically ill patients who needed mechanical ventilation for longer than two days, nebulized heparin was associated with a higher number of ventilator-free days. In line with results from preclinical studies, nebulization of heparin was found to have an effect on systemic coagulation, but without causing systemic bleedings.

**Conclusion:**

Local anticoagulant therapy through nebulization of anticoagulants attenuates pulmonary coagulopathy and frequently also inflammation in preclinical studies of lung injury. Recent human trials suggest nebulized heparin for ALI to be beneficial and safe, but data are very limited.

## Introduction

**Figure 1:**
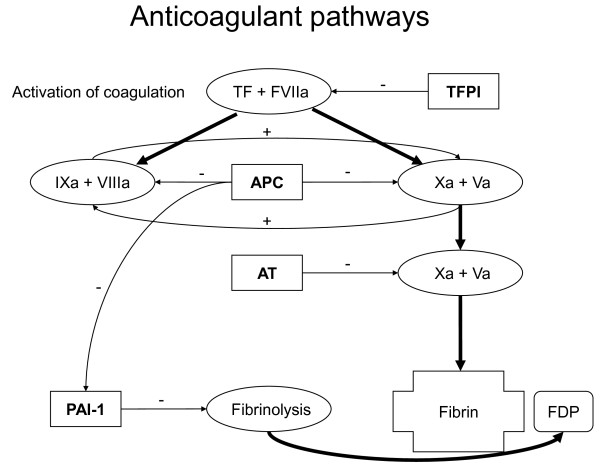
**Schematic and simplified presentation of coagulation, fibrinolysis and anticoagulant pathways**. The coagulation cascade is started through activation of tissue factor (TF)-factor VII (FVIIa) complex. Several coagulation factors accelerate the conversion of prothrombin to thrombin. Activated protein C (APC) can inactivate coagulation factors Va and VIIIa. Antithrombin (AT) serves to block the action of multiple coagulation factors (for example, Xa and IIa). Tissue factor pathway inhibitor (TFPI) inhibits stepwise the activation of coagulation factors. The fibrinolytic system is designed to degrade clots and fibrin degradation products (FDP) are formed. The main inhibitor of the plasminogen activators is plasminogen activator inhibitor type 1 (PAI-1). +: stimulating effect; -: inhibiting effect. Adapted and modified from Tuinman *et al. *[59].

Pulmonary coagulopathy is intrinsic to acute lung injury (ALI). Indeed, both microvascular thrombi and alveolar fibrin depositions are hallmarks of ALI, irrespective of its cause [1-5]. The extent of pulmonary coagulopathy depends on the severity of ALI [1] and is clearly linked to the outcome of ALI [6-9]. Pulmonary coagulopathy with ALI resembles systemic coagulopathy with sepsis [4], and is characterized by activated coagulation, attenuation of fibrinolysis, and enhanced breakdown and/or decreased production of natural anticoagulants (Figure 1) [4,10,11]. Extensive cross-talk between coagulation and inflammation may further inflame the lungs [3]. Indeed, activated coagulation factors may initiate or exaggerate injury [12-14], impairing alveolar aeration and perfusion [15], and promoting fibrosis [16].

Clinical trials inconsistently suggest beneficial effects of systemic anticoagulants in patients with ALI. Results from the PROWESS trial suggested patients with a pulmonary cause of their sepsis to benefit more from systemic anticoagulation with recombinant human (rh)-activated protein C (APC) than patients with sepsis from another source [17-19]. A recent clinical trial of patients with ALI even showed decreased pulmonary dead-space fraction with infusion of rh-APC, although this was not associated with an improved clinical outcome [20]. Notably, rh-APC has been withdrawn from the market, as the recent PROWESS-SHOCK trials showed no benefit from rh-APC in patient with septic shock [21]. Neither infusion of antithrombin (AT) nor infusion of rh-tissue factor pathway inhibitor (TFPI) has been found to improve outcome in sepsis [22,23]. Infusion of AT, however, was found to prevent new pulmonary dysfunction [24]. Infusion of rh-TFPI was suggested to improve survival of patients with a community-acquired pneumonia [25] but failed to improve outcome of patients with severe community-acquired pneumonia in the recent CAPTIVATE trial [26]. Finally, post hoc analysis of four large clinical trials of patients with sepsis suggested infusion of low-dose heparin to improve survival, although unintended pitfalls may not have been accounted for [27]. However, one recent clinical trial showed no effect on survival with infusion of unfractioned heparin [28].

High pulmonary concentrations of an anticoagulant may be necessary to have any effect on pulmonary coagulation, maybe higher than achievable with systemic treatment. Similar to systemic antimicrobial therapy for pneumonia, with which antimicrobial agents penetrate in dissimilar amounts into the lung [29], it could be argued that anticoagulants may not penetrate into lung tissue adequately to have a local effect. A major concern is the association between systemic anticoagulation and the high incidence of serious bleeding complications in patients with sepsis, which at times could be life-threatening [17,22,23,30,31]. With higher systemic dosages, possibly necessary to have sufficiently high concentrations in the lung, without doubt the incidence of bleedings would rise.

Local administration of anticoagulants, through nebulization, may allow for higher pulmonary concentrations while, at the same time, preventing systemic bleedings. To test the hypothesis whether nebulization of anticoagulants attenuates pulmonary coagulation without having systemic side-effects, we searched the literature for preclinical studies and human trials of nebulized anticoagulants in the context of lung injury in animals and ALI in humans. We aimed to search for any beneficial and harmful effects of nebulized anticoagulants.

## Materials and methods

### Data sources

To identify relevant manuscripts on local anticoagulation in the setting of lung injury in animals and ALI in humans, two search strategies were followed. First, an electronic search in Medline and Embase databases was conducted. Second, the reference lists of retrieved papers were screened for potentially important papers. The search was limited to papers published from 1980 until now, and papers written in the English language.

### Keywords

**Figure 2:**
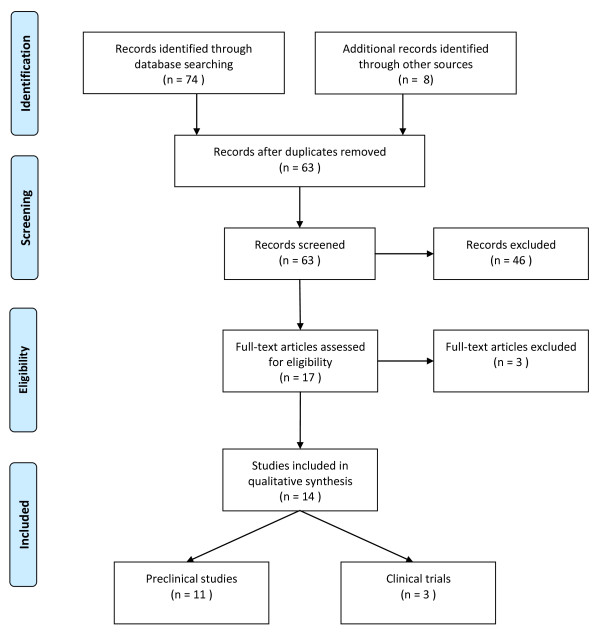
**Prisma flow diagram showing the search strategy and selection process**.

The Medline database was used to identify medical subject headings (MeSH) to select search terms. In addition to MeSH terms, also free text words were used. Search terms referred to aspects of the condition ('acute lung injury' (ALI), 'acute respiratory distress syndrome' (ARDS)) as well as related conditions ('pneumonia', sepsis', and 'ventilator-induced lung injury' (VILI)). In addition, we searched on the intervention ('nebulized', 'vaporized', and 'aerosolized'), and anticoagulant agents ('APC', 'AT', 'TFPI', 'heparin' and 'danaparoid'). We chose to limit the search of agents to those that have been tested in phase III trials of sepsis (rh-APC, AT and rh-TFPI), and those commercially available and frequently given to critically ill patients (heparin and danaparoid). The same search strategy was used in Embase. A Prisma flow diagram of the applied search strategy and selection process is summarized in Figure 2.

### Study selection

Titles and abstracts of identified manuscripts were reviewed on:

• Population (that is, animal models of lung injury or patients with ALI) and the related settings (pneumonia, sepsis or VILI)

• Intervention (that is, local anticoagulation)

• Outcome (that is, lung injury and inflammation, pulmonary coagulation, systemic coagulation and bleeding)

In case of uncertainty, the complete manuscript was obtained and evaluated.

### Data extraction

Manuscripts were critically appraised along two research questions:

• Do nebulized anticoagulants affect pulmonary coagulation, parameters of lung injury, and/or outcome?

• Do nebulized anticoagulants affect systemic coagulation and, as such, cause bleedings?

## Results

### Preclinical studies of local anticoagulation

The search of preclinical studies of pulmonary anticoagulation yielded 10 preclinical studies of nebulized and one with intratracheal anticoagulants for lung injury with a large diversity in outcome measures (Table 1). Four preclinical studies tested nebulized rh-APC [32-35], four studies compared rh-APC, plasma-derived human AT, heparin and danaparoid [36-39], one study tested heparin [40], one study compared nebulized heparin with systemic infusion of heparin [41], and one study compared nebulized heparin with systemic infusion of heparin and lisofylline (LSF) [42]. The search did not yield any preclinical study that investigated the effects of nebulized rh-TFPI.

**Table 1:** Animal models of acute lung injury evaluating the effects of treatment with local anticoagulant therapy.

Drug (dose)	Animal	Lung injury model and nebulizer	Effect parameter, observed effect and safety	Reference
Rh-APC (12.5 μg/h)	mice	Mechanical ventilation Aeroneb^a^	Rh-APC attenuated pulmonary inflammation, improved oxygenation, and prevented endothelial dysfunction. Rh-APC did not increase pulmonary bleeding.	Maniatis [32]

Rh-APC (48 μg/kg/h)	sheep	i.v. LPS Servo Ultra^b^	Rh-APC improved oxygenation and increased aerated lung volume; EVLW was unaffected. Rh-APC did not cause systemic bleeding.	Waerhaug [33]

Rh-APC (2 × 25 or 100 μg) and repeated dosing	mice	i.t. LPS Aeroneb Pro^a^	Rh-APC attenuated pulmonary coagulation and inflammation; Rh-APC improved lung function. No differences between single and repeated dosing. Effects on systemic coagulation or systemic bleeding were not reported.	Slofstra [34]

Rh-APC (4 mg/3 mL)	mice	i.t. LPS DeVilbiss^c^	Rh-APC attenuated pulmonary inflammation, decreased VCAM-1 upregulation and prevented changes in histopathology. Effects on systemic coagulation or systemic bleeding were not reported.	Kotanidou [35]

Rh-APC (5,000 μg/kg) Heparin (1,000 U/kg) Plasma-derived human AT (500 IU/kg) Danaparoid (250 E/kg)	Rats	*S. pneumoniae *pneumonia Aeroneb Pro^a^	All agents attenuated pulmonary coagulation. Plasma-derived human AT also attenuated pulmonary inflammation, bacterial outgrowth and changes in histopathology. Only danaparoid affected systemic coagulation. Systemic bleeding was not reported	Hofstra [36]

Rh-APC (5,000 μg/kg) Heparin (1,000 U/kg) Plasma-derived human AT (500 IU/kg) Danaparoid (250 E/kg)	Rats	i.v. LPS Aeroneb Pro^a^	All agents attenuated pulmonary coagulation; pulmonary inflammation and histopathology were not affected. Heparin and danaparoid affected systemic coagulation. Systemic bleeding was not reported	Hofstra [37]

Heparin (5 μg) i.t.	mice	*Legionella* pneumonia No nebulizer used	Heparin improved survival and decreased pulmonary inflammation, bacterial outgrowth, *Legionella *adherence and endothelial permeability. Effects on systemic coagulation or systemic bleeding were not reported.	Ader [40]

Heparin (10,000 IU/4 h) AT (290 IU) or a combination	sheep	burn and smoke inhalation Nebulizer not stated	Combination therapy improved hemodynamics and P/F ratio; airway obstruction and wet-to-dry weight decreased. Systemic clotting time was unaffected. Systemic bleeding was not reported.	Enkhbaater [38]

Combination therapy of Heparin (10,000 IU/4 h) and plasma-derived human AT i.v. (0.34 mg/kg/h)	sheep	burn and smoke inhalation Nebulizer not stated	Heparin + plasma-derived human AT improved P/F ratio; central venous pressure, airway obstruction and wet-to-dry weight decreased. Systemic levels of AT were elevated. Systemic bleeding was not reported.	Enkhbaatar [39]

Heparin (10,000 IU/4 h) or Heparin i.v. (5,300 U/kg/23 h)	sheep	burn and smoke inhalation + *P. aeruginosa *pneumonia Airlife Misty^d^	Nebulization of heparin improved hemodynamics and P/F ratio; airway obstruction, wet-to-dry weight and changes in histopathology were decreased. Systemic clotting time was unaffected with nebulized heparin. Systemic bleeding was not reported.	Murakami [41]

Heparin (10,000 IU/4h) and/or Lisofylline i.v. (10 mg/kg/h after bolus 20 mg/kg)	sheep	burn and smoke inhalation Nebulizer not stated	Combination therapy decreased the need of mechanical ventilation, P(A-a)O_2 _and pulmonary shunt fraction; wet-to-dry weight and changes in histopathology were unaffected. Effects on systemic coagulation and systemic bleeding were not reported.	Tasaki [42]

Drugs delivered intravenously (i.v.) and intratracheally (i.t.) are described in the table See original manuscript for details. ^a^Aerogen, Galway, Ireland. ^b^Siemens-Elema AB, Solna, Sweden. ^c^Health Care Worldwide, Somerset, PA, USA. ^d^Allegiance Healthcare, McGaw Park, IL, USA. AT, antithrombin; EVLW, extravascular lung water; LPS, lipopolysaccharide; P/F, PaO2/FiO2; Rh-APC, recombinant human-activated protein C; VCAM-1, vascular cell adhesion molecule 1

#### Recombinant human-activated protein C

Although in none of the preclinical studies pulmonary levels of rh-APC were measured, nebulization of rh-APC was found to attenuate activation of pulmonary coagulation [34,36,37], and to stimulate pulmonary fibrinolysis [36]. Nebulization of rh-APC inconsistently reduced pulmonary inflammation [34-36]. Furthermore, nebulization of rh-APC was found to improve oxygenation [32,34,35], and to reduce histolopathological derangements [32,34,35]. While nebulization of rh-APC improved aerated lung volume, it had no effect on extravascular lung water (EVLW) [33]. Nebulization of rh-APC has neither been associated with systemic coagulation [33] nor with signs of systemic bleeding [36]. Dosages and timing of rh-APC varied amongst studies: from five times 48 μg/kg [33] up to 200 mg/kg as a single dose [35].

#### Plasma-derived human antithrombin

Nebulization of plasma-derived AT was found to increase pulmonary levels of AT [36,37], to attenuate activation of pulmonary coagulation [36,37] and to stimulate pulmonary fibrinolysis [36]. Alike with rh-APC, nebulized plasma-derived AT inconsistently reduced pulmonary inflammation [36,38]. Furthermore, nebulization of plasma-derived AT was found to reduce histopathological derangements and bacterial outgrowth in models of infectious lung injury [36]. Nebulization of plasma-derived AT has not been associated with an affect on systemic coagulation [36,37]. Dosages of plasma-derived AT varied amongst studies: from 10 IU/kg [38] to as high as 500 IU/kg [36,37].

#### Heparin

Nebulization of heparin and intratracheal-installed heparin were found to attenuate pulmonary coagulopathy [36,37,40]. Nebulization of heparin, as expected, was not found to affect fibrinolysis [36,37,40]. Intratracheal-installed heparin reduced pulmonary inflammation and endothelial permeability. In addition intratracheal-installed heparin improved survival, reduced bacterial outgrowth and bacterial adherence to lung epithelium [40]. Nebulization of heparin did not reduce pulmonary inflammation [37]. Furthermore, nebulization of heparin did affect systemic coagulation [37]. The dosages of heparin varied between an estimated 333 IU/kg [38,39,41,42] and 1000 IU/kg [36,37].

#### Danaparoid

Nebulization of danaparoid was also found to attenuate pulmonary coagulopathy but not fibrinolysis [36,37]. Danaparoid reduced local coagulation, but not inflammation [37]. Danaparoid was found to have an effect on systemic coagulation [36,37]. A dose of 250 E/kg was used in both studies.

#### Combined therapies

Combined nebulization of AT or systemic infusion of AT with nebulization of heparin was found to reduce wet-to-dry lung weight ratios and airway obstructions, and to improve lung function [38,39]. Combined nebulization of heparin and systemic lysofylline was found to reduce need for mechanical ventilation, to decrease pulmonary shunt fraction, but not to reduce wet-to-dry lung weight ratios [42]. Combined nebulization of heparin and AT was not found to affect clotting times [38,39].

### Clinical trials of nebulized anticoagulants

The search did not yield any clinical trial that investigated the effects of nebulization of rh-APC, plasma-derived human AT or rh-TFPI; three trials tested the nebulization of heparin (Table 2).

**Table 2:** Human models of acute lung injury evaluating the effects of treatment with nebulized anticoagulant therapy.

Drug	Model, design, dose and nebulizer	Effect parameter, observed effect and safety	Reference
Heparin	RCT of 50 patients expected to need > 48 hours of MV 25,000 U 6 dd for 14 days Aeroneb Pro^a^	Heparin increased the number of ventilator-free days; P/F ratio and mortality were unaffected. There was a trend for less tracheotomies with heparin. The incidence of systemic bleeding was not affected; heparin increased APTT.	Dixon [15]

Heparin + N-acetylcysteine + albulterol	Case control study of 30 patients after smoke inhalation 10,000 U 6 dd for 7 days Nebulizer not stated	Heparin + acetylcysteine + albulterol improved survival and lung injury score. Effects on systemic coagulation and systemic bleeding were not reported.	Miller [45]

Heparin	Phase 1 trial of 16 patients with ALI 50-400 × 10^3 ^U/day for 2 days Aeroneb Pro^a^	Heparin attenuated pulmonary coagulation; P/F ratio and lung compliance were not affected. Heparin increased APTT. The incidence of systemic bleedings was not affected.	Dixon [43,44]

See original manuscript for details. ^a^Aerogen, Galway, Ireland. ALI: acute lung injury; APTT, activated partial thromboplastin time; MV, mechanical ventilation; RCT, randomized controlled trial.

#### Heparin

In a phase I trial of patients with ALI, heparin was nebulized at different dosages, namely 50,000 U/day, 100,000 U/day, 200,000 U/day or 400,000 U/day for two days. An Aeroneb Pro (Aerogen, Galway, Ireland) vibrating mesh nebulizer was used in this study. Sixteen patients were enrolled with a mean age of 58 years. Patients were ventilated in a pressure-support mode of ventilation and peak airway pressures were maintained below 35 cm H_2_O. Nebulization of heparin partly prevented activation of coagulation, evidenced by a reduction of thrombin-antithrombin complexes and fibrin degradation products in the broncheoalveolar lavage fluid, but lung function remained unchanged. With higher dosages, nebulization of heparin was found to be associated with an increase of systemic activated partial thromboplastin time (APTT) [43,44], without causing systemic bleedings.

In a single-center, retrospective case-control study of 30 patients with smoke inhalation-associated ALI, cases were compared with historical controls. All patients were ventilated with volume-cycled ventilation at a tidal volume of 5 to 8 ml/kg. The peak airway pressures were maintained below 40 cmH_2_O. The dose of nebulized heparin used was 10,000 U every four hours for seven consecutive days. Nebulization of heparin combined with N-acetylcysteine and albuterol sulfate was associated with an improved survival and improved lung injury scores (LIS) [45]. No data on pulmonary coagulation or inflammation and nebulizer used were reported.

In a randomized controlled trial of 50 patients who were expected to require mechanical ventilation for more than 48 hours' treatment with 25,000 U of heparin for six times a day, with a maximum of 14 days, was compared to placebo [15]. An Aeroneb Pro vibrating mesh nebulizer was used in this study. Of note, pulmonary levels of coagulation and inflammation were not affected. No difference in bleeding events was observed between groups, even in patients with co-administrated systemic heparin, but APTT levels were higher in the intervention group. No effect on mortality was seen, but treatment with heparin was associated with fewer days of mechanical ventilation (MV) and a trend for a higher PaO2/FiO2 (P/F) ratio.

## Discussion

Ten years ago, to many in the pulmonary or critical care community the idea that anticoagulants could be a beneficial therapy in ALI may have been surprising and the approach unfamiliar [46]. Nowadays, several preclinical and clinical trials have been conducted with anticoagulants in the setting of experimental lung injury in animals or ALI in humans. The clinical evidence for pulmonary anticoagulant therapy is severely limited, since only one randomized controlled trial has been performed. The identified preclinical studies all showed clear evidence for attenuating effects of nebulized anticoagulants on local coagulation, a phenomenon that has been associated with morbidity and mortality of ALI. Given the extensive crosstalk between inflammation and coagulation [47], the observed diminishing effects on local coagulation may be countable for dampened pulmonary inflammation with nebulization of anticoagulants.

It should be noted that while rh-APC and rh-TFPI were infused in a continuous fashion in clinical trials of sepsis [17,23,30,31,48,49], in the reviewed preclinical studies these anticoagulants were nebulized intermittently. Although half-life of rh-APC in plasma is short [50], rh-APC could be detected in mice bronchoalveolar lavage fluid up to 24 hours after inhalation [51]. It is uncertain whether continuous nebulization would improve the pulmonary effects of rh-APC, but considering longer bioavailability and intermittent dosing, local administration may affect both efficacy and costs.

Nebulization of rh-APC or plasma-derived human AT did not affect systemic coagulation, suggesting local administration of these agents to be safe and, as such, to have a potential benefit over systemic administration. Nebulization of heparin or danaparoid, however, did affect systemic parameters of coagulation. These agents seem to have the potential to leak from the pulmonary compartment into the circulation [36,37]. This may not come as a surprise, since both heparin and danaparoid are relatively small molecules (8 and 5.5 kDa respectively), much lower than APC and AT (56 and 58 kDa respectively). Of note, in a phase I trial, nebulization of heparin in a high dosages lengthened clotting time only after > 24 hours [43], suggesting that heparin may be stored and metabolized in pulmonary endothelial cells, initially limiting heparin from reaching the systemic circulation. Notably, the results from the clinical trials of nebulization of heparin showed no increase on systemic bleedings.

Five major variables influence local drug delivery in the lung during mechanical ventilation, that is, the aerosol generator, aerosol particle size, conditions in the ventilatory circuit, artificial airway and ventilator parameters [52]. Unfortunately, most of these variables were insufficiently addressed in the retrieved studies. In addition, different types and brands of nebulizers were used, including jet [35,41], ultrasonic [33] and vibrating mesh nebulizers [34,36,37]. Some studies did not report which nebulizer was used [38,39,42]. No studies mention the particle size delivered by the used nebulizer and probable losses of study drug, which would have been of interest, since it is one of the determinants of drug delivery to the lungs [52]. In addition, maldistribution of nebulized drugs to areas of better ventilation away from areas of collapse and consolidation is a concern of nebulized agents. After nebulization, uptake through the bronchial mucosa and distribution by a rich network of submucosal capillaries to other areas of the lung, could lead to adequate concentrations at various sites in the lung, as seen with inhaled antibiotics [53]. Another potential risk is that with higher local concentrations of anticoagulants in the lung, systemic measures of anticoagulation may not measure local bleeding risk. These aspects need further attention in future studies.

A potential harm from local anticoagulant therapies lies in the fact that part of the procoagulant response may be needed for healing. Notably, coagulation and the formation of fibrin are natural responses to injury. The coagulation system may play a role in containing pathogens to the side of infection [54]. In a model of *Pseudomonas *pneumonia, interfering with the initial procoagulant response showed to be a potentially dangerous strategy, since it was associated with increased bacteremia [55,56]. In the studies identified by our search, no increased spread of bacteria was observed, though. On the other hand, anticoagulants may also have beneficial antimicrobial effects. Indeed, local heparin has been shown to have an anti-adherence effect, making it, for *Legionella pneumophila*, unable to adhere to airway epithelial cells in the alveolar space [40]. Also, AT has been shown to limit bacterial outgrowth of *Streptococcus pneumoniae *in a rat model of pneumonia with this pathogen [36]. Thus, anticoagulants could also act as an adjuvant to conventional antimicrobial therapy in patients who are mechanically ventilated.

Our search identified only three trials of local anticoagulants in patients [15,43,45]. The trial of patients who needed prolonged mechanical ventilation knows several potential weaknesses. The population was very heterogeneous, since it included unselected patients requiring mechanical ventilation beyond 48 hours, independent of the reason. Furthermore, a large proportion of patients received systemic heparin (24 versus 32%, in heparin-treated and control patients respectively). Finally, it should be mentioned that this trial was performed in a single ICU.

Investigations in animals and patients with burns and smoke inhalation and burns are a distinct category, since this insult is characterized by massive airway obstructive casts [38], resulting in atelectasis, air trapping, alveolar hyperinflation and barotraumas, and maybe even pneumonia [39,57,58]. The effects of nebulized anticoagulants on cast formation may not be comparable to the anticoagulant effects of these agents in ALI by another cause. One major weakness of the clinical trial that showed beneficial effects of nebulized heparin in these patients is that historic controls were used [45]. Furthermore, side-effects were not reported.

Due to the marked differences in study design and patient characteristics, the cumulative summary of these data should be interpreted with caution. But this preliminary analysis, despite a number of caveats, suggests further studies to be undertaken. We are in need of more animal studies and clinical trials that address the promising concept of administration of anticoagulants to the lungs. We favor mechanistic studies before randomized controlled trials for several reasons. First, dosages were arbitrarily chosen in most preclinical studies. Second, assessment of distribution of inhaled anticoagulants in the lungs and its final (plasma) concentrations have to be better addressed. While larger agents may have limited access to the lower airways, smaller agents may leak more easily into the systemic circulation. Third, animal models of pneumonia should mimic as much as possible the clinical scenario and as such, should include, for instance, the use of antibiotics.

## Conclusions

Preclinical studies in models of lung injury show nebulization of anticoagulants to reduce pulmonary coagulopathy and inflammation. Neither nebulized APC nor nebulized AT affects systemic coagulation, whereas heparin and danaparoid do affect systemic coagulation. Initial clinical trials of nebulized heparin show beneficial effects in ALI.

## Key messages

• Nebulization of anticoagulants attenuates pulmonary coagulopathy and pulmonary inflammation

• Neither nebulized APC nor nebulized AT affect systemic coagulation, whereas heparin and danaparoid do affect systemic coagulation

• Nebulization of heparin improves survival in patients with smoke inhalation-associated ALI

• Nebulization of heparin reduces the days of mechanical ventilation in patients with prolonged need for mechanical ventilation

## Abbreviations

ALI: acute lung injury; APC: activated protein C; APTT: activated partial thromboplastin time; ARDS: acute respiratory distress syndrome; AT: antithrombin; EVLW: extravascular lung water; i.t.: intratracheal; i.v.: intravenous; LIS: lung injury score; LSF: lisofylline; LPS: lipopolysaccharide; MV: mechanical ventilation; MeSH: medical subject headings; P/F: PaO2/FiO2; rh: recombinant human; RCT: randomized controlled trial; TATc: thrombin-antithrombin complex; TF: tissue factor; TFPI: tissue factor pathway inhibitor; VCAM-1: vascular cell adhesion molecule 1; VILI: ventilator-induced lung injury.

## Competing interests

The authors declare that they have no competing interests.

## Authors' contributions

PRT was intimately involved in data gathering, interpretation of the results as well as manuscript preparation. He has read the final version of the manuscript and agrees with all reported findings and interpretations. BD was intimately involved in interpretation of the results and manuscript. He has read the final version of the manuscript and agrees with all reported findings and interpretations. ML and NPJ are members of an expert panel dealing with coagulation and coagulation disorders in critically ill patients. They were instrumental in study hypothesis. They were intimately involved with interpretation of the results and manuscript. They have read the final version of the manuscript and agree with all reported findings. MJS was instrumental in study design, data analysis and preparing the manuscript. He has read the final version of the manuscript and agrees with all reported findings and interpretations.

All authors have approved the final manuscript.
